# Genetic architecture of limit dextrinase inhibitor (LDI) activity in Tibetan wild barley

**DOI:** 10.1186/1471-2229-14-117

**Published:** 2014-05-01

**Authors:** Yuqing Huang, Shengguan Cai, Lingzhen Ye, Yong Han, Dezhi Wu, Fei Dai, Chengdao Li, Guoping Zhang

**Affiliations:** 1 Agronomy Department, Key Laboratory of Crop Germplasm Resource of Zhejiang Province, Zhejiang University, Hangzhou 310058, China; 2 Department of Agriculture and Food, Western Australia, WA 6983, Australia

**Keywords:** Limit dextrinase inhibitor (LDI), Genome-wide association study (GWAS), Single nucleotide polymorphism (SNP), Tibetan wild barley

## Abstract

**Background:**

Limit dextrinase inhibitor (LDI) inhibits starch degradation in barley grains during malting because it binds with limit dextrinase (LD). There is a wide genetic variation in LDI synthesis and inactivation during barley grain development and germination. However, the genetic control of LDI activity remains little understood.

**Results:**

In this study, association analysis was performed on 162 Tibetan wild accessions by using LDI activity, 835 Diversity Arrays Technology (DArT) markers and single nucleotide polymorphisms (SNPs) of the gene *HvLDI* encoding LDI. Two DArT markers, bpb-8347, bpb-0068, and 31 SNPs of *HvLDI* were significantly associated with LDI activity, explaining 10.0%, 6.6% and 13.4% of phenotypic variation, respectively. Bpb-8347 is located on chromosome 6H, near the locus of *HvLDI*, and bpb-0068 is located on 3H.

**Conclusions:**

The current results confirmed the locus of the gene controlling LDI activity and identified a new DArT markers associated with LDI activity. The SNPs associated with LDI activity may provide a new insight into the genetic variation of LDI activity in barley grains.

## Background

Malting is the first step in brewing or beer production of malt barley, and the properties of malting barley are generally evaluated on the basis of malting quality. High malting quality is characterized by high malt extract (ME) and diastatic power (DP), as well as high wort fermentation by yeast, i.e. complete degradation of starch and polysaccharide in malt [1]. The major chemical compound in barley grains is starch, which consists of 30% amylose and 70% amylopectin [2].

Starch is degraded by coordinative action of α-amylase, β-amylase, limit dextrinase (LD) and α-glucosidase in the endosperm of the germinating grains [3]. LD is the only enzyme of cleaving α-1-6 linkages in branched dextrins molecules [4]. Without the action of LD, the branched dextrins could not be fermented by yeast [5]. Hence, LD plays an important role in the brewing and distilling industries for producing fermentable sugar.

LD in barley grains exists in three different forms: insoluble bound, soluble inactive and active free, and only the active free form participates in starch mobilization [6]. However, the free LD accounts for a small portion of total LD. The low activity of available LD in grains is due to its combination with endogenous inhibitors, limit dextrinase inhibitor (LDI) [7]. LD and LDI interact with each other in a 1:1 molar ratio complex [8]. The LD bound to LDI is thought to be a limiting factor for complete degradation of starch [9].

LDI is synthesized in the developing grains, and degraded gradually during the malting process [7]. Consequently, LD activity in malt is not only dependent on the amount of the enzyme itself in mature grains, and also is closely related to the amount and type of LDI. *HvLDI* was identified to encode the LDI protein, and located at chromosome 6H [10,11]. However, genetic variation and controlling of LDI synthesis and degradation in barley grains and malt is not totally understood. In view of a wider variation of LD activity in Tibetan wild barley than in cultivated barley (unpublished data), it is imperative to determine the genetic variation of LDI content in Tibetan wild barley, which has been recently proved to be a progenitor of cultivated barley and is rich in genetic diversity [12,13].

Association analysis has been widely used to identify genes or loci of important traits, such as abiotic stress tolerance [14,12,15], agronomic traits [16,17] and also barley quality [18,19]. However, there has been no report on the discovery of novel loci or elite alleles attributed to LDI activity using association analysis.

The objectives of this study were to (1) determine the genetic variation of LDI activity in Tibetan wild barley; (2) identify the DArT markers and SNPs of *HvLDI* associated with LDI activity in Tibetan wild barley.

## Methods

### Plant materials

A total of 162 Tibetan wild barley accessions, kindly provided by professor Sun of Huazhong Agricultural University, China, were used for LDI activity and association analysis. All accessions were planted in early November 2011 and 2012 in Zijingang campus, Zhejiang University (Hangzhou, China). Every genotype was cultivated in accordance with local agronomic practices with three replications. The 162 barley accessions were planted in a block with each accession consisting of three lines (2 m length and line distance is 0.25 m).

### Assay of LDI activity

Grain samples were micro-malted in a Phoenix System Micro-malting Apparatus (Adelaide, Australia) in the order of steeping, germination and drying. LD was partially purified from barley malt according to Kristensen et al. [20]. The fractions were applied to gel filtration chromatography after the ion exchange chromatography step, and partially purified LD was collected and used for the measurement of LDI activity.

LDI activity was determined according to MacGregor et al. [21] with some modification. One ml of 0.1 M sodium acetate (pH 5.5) containing 10 mM 1,10-phenanthroline was added to 0.1 g barley powder, incubated at 4°C for 30 min. The extract was heated at 70°C for 40 min, centrifuged and the supernatant was collected. The protein content of the extracts was measured using Bradford assay kits (Sangon Biotech). Twenty micrograms protein of LDI extract were mixed with 10 mU partially purified LD and the volume was made up to 0.5 ml in 0.1 M maleic acid containing 0.02% Na azide (pH 5.5). The remaining LD was determined using the Limit-Dextrinase assay kit (Megazyme). The LDI activity was calculated as the reduced LD activity.

### Population structure and kinship analysis

Totally, 771 DArT markers, with minor allele frequency (MAF) higher than 0.03 (Additional file 1: Table S1), were used for population structure analysis using STRUCTURE software (v2.3.3) [22], in which the number of clusters (k) was set from 1 to 12 and ten iterations were performed in an admixture model with 10,000 burning period and 100,000 MCMC (Markov Chain Monte Carlo). The DArT markers used were derived from Diversity Arrays Technology Pty Ltd, Australia and distributed over the whole genome [23-25]. The most probable number of clusters (k) was estimated according to the value of Δk. When Δk had the highest value, the value of k was the number of clusters [26]. Kinship was estimated using SPAGeDi software [27]. Genetic distance and neighbor-joining tree were developed with NTSYSpc (version 2.10e).

### Variance component and heritability estimation

Variance analysis was conducted using SPSS software. The used model was: y = mu + GENOTYPE + ERROR (fixed in low case, random in capitals). Heritability was estimated according to H^2^ = Vg/(Vg + Ve).

### Genome-wide association study (GWAS) of LDI activity

Genome-wide association study between LDI activity (mean value of 2011 and 2012) and DArT markers was conducted using TASSEL software (v3.0), where Q, K and Q + K methods were applied [28]. The structure matrix was included as covariate to correct population structure in Q model: *y* = *Qν* + *Xβ* + *e*, where *X* is the DArT marker matrix, *Q* is the structure matrix, *β* and *ν* are coefficient vectors for DArT marker vector and population structure vector, and e is the random error vector. In K model: *y* = *Xβ* + *Zu* + *e*. The Q + K model can be written in a matrix form as: *y* = *Qν* + *Xβ* + *Zu* + *e*, where *X*, *β*, *Q* and *ν* are the same as those mentioned above, *Z* is the kinship matrix, and *u* is a vector of random genetic effects u ~ N (0, 2 K), where K is the kinship matrix. Manhattan plots were displayed using R software (v2.14.2).

### PCR amplification and sequencing

Genomic DNA was extracted from leaves of 150 barley seedlings using CTAB method [29]. The primers used for DNA amplification were designed using the primer design tool of NCBI (http://www.ncbi.nlm.nih.gov/tools/primer-blast/). Primers for *HvLDI* were: forward, TTTTCGCATGTCACCAAAAATGT; reverse, TCCGCTTCATTACCTTGGCG. The amplified DNA fragment was about 900 bp.

The PCR reactions were completed as follows: 2.5 μl of 10 × TransTaq HiFi buffer I (200 mM Tris–HCl (pH 8.4), 100 mM (NH_4_)_2_SO_4_, 20 mM MgCl_2_, 200 mM KCl, 2 μl of 2.5 mM dNTPs, 1 μl of 10 μM forward primers, 1 μl of 10 μM reverse primer, 0.5 μl of 5 units μl^-1^ of TransTaq polymerase High Fidelity (Beijing TransGen Biotech Co., Ltd.), and 1 μl of 50 ng of genomic DNA. The PCR amplification program started at 5 min at 95°C, followed by 32 cycles of 30 s at 95°C, 30 s at 60°C, and 1 min at 72°C, and then 10 min at 72°C for a final extension. The PCR products were analyzed by 1% agarose gel electrophoresis. They were sequenced using 3730 × l DNA Analyzer (Applied Biosystems Inc., USA).

### Polymorphism of HvLDI and haplotype analysis

Alignment and polymorphism detection of *HvLDI* sequences were analyzed by MEGA v5.2 [30]. The haplotype analysis was conducted with Dnasp v5.0 [31].

### Availability of supporting data

The sequences of the 6 haplotypes were deposited in GenBank, and the accession numbers of these haplotypes, H1-H6 are KJ710426, KJ710427, KJ710427, KJ710429, KJ710430 and KJ710431, respectively.

## Results

### LDI activities of 162 Tibetan wild accessions

**Figure 1:**
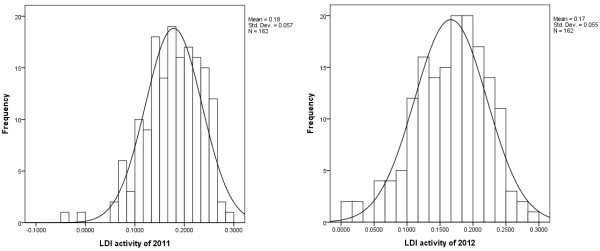
**The distribution frequency of LDI activity of the examined Tibetan wild barley accessions in 2011 and 2012.** The X axis represents LDI activity, the Y axis represents frequency of LDI activity.

A wide genetic variation of the LDI activity was observed among 162 Tibetan barley accessions for two years (Figure 1), ranged from -0.034 mU/μg to 0.295 mU/μg for 2011 and from 0.003 mU/μg to 0.287 mU/μg for 2012, with a mean of 0.176 mU/μg and standard difference (SD) of 0.057 for 2011 samples, and a mean of 0.167 mU/μg and SD of 0.055 for 2012.

The results of variance analysis are presented in Table 1. Estimates of the variance component for genotype and error were 0.00320 and 0.000248 for 2011, 0.00288 and 0.000493 for 2012. The heritabilities for 2011 and 2012 were 0.928 and 0.854, respectively.

**Table 1:** **Variance component and heritability** (**H**^**2**^) **estimates for LDI activity of 2011 and 2012**

	**Vg**	**Ve**	**H**^**2**^
Year 2011	3.20E-03	2.88E-04	0.928
Year 2012	2.48E-03	4.93E-04	0.854

Note: Variance components are shown for Genotype (Vg), Error (Ve).

### Population structure

**Figure 2:**
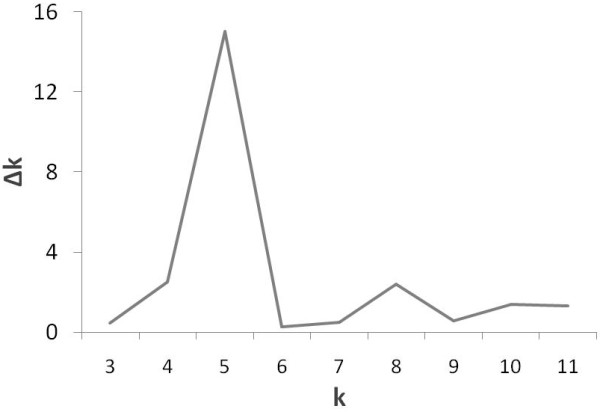
**Estimation of the most probable number of clusters (k), based on 10 independent runs and k ranging from 1 to 11.**

**Figure 3:**
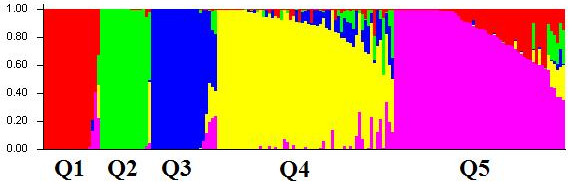
**Population structure of 162 Tibetan barley accessions.** Population structure of 162 barley accessions was divided based on genetic diversity detected by 835 DArT markers with k = 5. Five subpopulations were represented by different colors.

The largest value of statistic index Δk was used as an indicator of the most probable number of subpopulations for all accessions (Figure 2). In the present study, the 162 accessions could be classified into five subpopulations, with 19, 17, 21, 50 and 55 accessions for the each individual subpopulation (Figure 3). The result was consistent with the data from the cluster analysis (Additional file 2: Figure S1). The population structure of 162 Tibetan wild barley accessions was listed in Additional file 3: Table S2.

### Genome-wide association study

**Figure 4:**
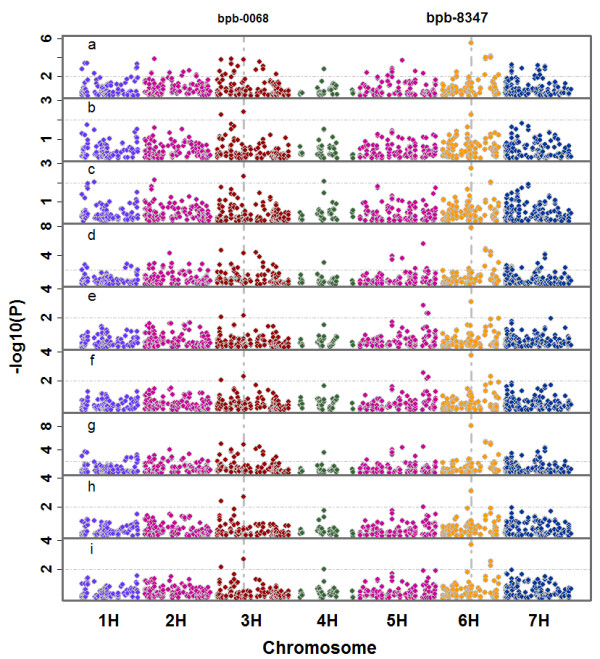
**GWAS of LDI activity of 162 Tibetan barley accessions.** GWAS was analyzed by three methods: a, d and g: Q method for 2011, 2012 and mean values of 2011 and 2012; b, e, h: K method for 2011, 2012 and mean values; b, d and f: Q + K method for 2011, 2012 and mean values. Significant associations were identified using criterion of -log_10_(P) >2 (P < 0.01).

The LDI activities of 162 accessions and 771 DArT markers were used to perform genome-wide association study (GWAS). Two markers, bpb-8347 and bpb-0068 showed significance under Q, K and Q + K methods (Additional file 4: Table S3). When Q method was used, the -log_10_(p) values for bpb-8347 and bpb-0068 were 5.48 and 3.75 in 2011 and 7.76 and 4.26 in 2012. If only kinship was considered, the -log_10_(p) values for bpb-8347 and bpb-0068 were 2.40 and 2.25 in 2011, and 3.10 and 2.15 in 2012. When a more restrict method, Q + K method was applied, the -log_10_(p) values were 2.75 and 2.30 in 2011, and 3.74 and 2.29 in 2012, all of which reached the threshold of significance in association analysis: -log10(P) = 2 (Figure 4). For the mean LDI activity of the two years, the -log_10_(p) values for bpb-8347 and bpb-0068 were 8.07 and 4.88, respectively, if considered from only one population structure, and 3.06 and 2.66 if only kinship being considered, and 3.70 and 2.71, if both population structure and kinship being considered.

Bpb-8347 and bpb-0068, located at 73.44 cM on chromosome 6H and 66.50 cM on 3H, showed significant associations with LDI activity, explaining 6.8% and 5.3% of phenotypic variation in 2011 and 10.4% and 5.5% in 2012 under Q + k method. Similarly, the two DArT markers could explain 10.0% and 6.6% of phenotypic variation for the mean values of 2011 and 2012.

### Association between HvLDI and LDI activity and haplotype analysis

**Figure 5:**
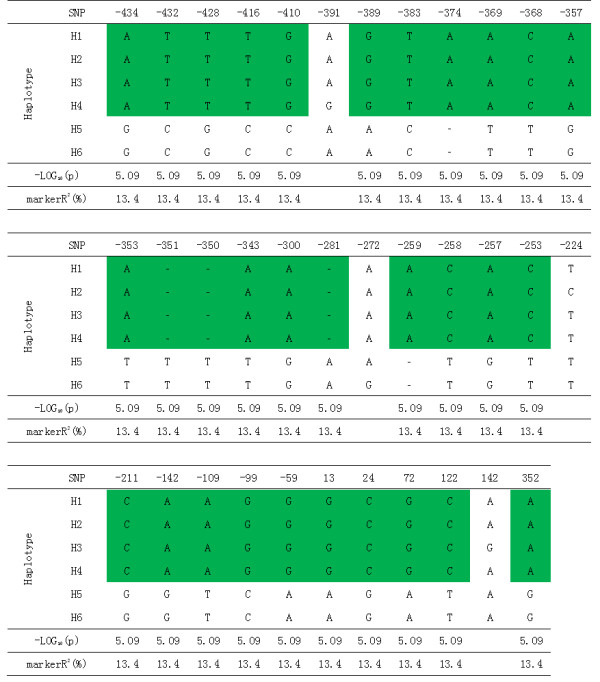
**Haplotype analysis of the *HvLDI *using 35 SNPs.** The first site of coding region was presented as 0. The green areas are the SNPs significantly correlated with LDI activity, for which the value of -log_10_(P) >2. Marker R^2^ is the fraction of the total variation explained by the marker.

The variation of *HvLDI* and its promoter region were further investigated because several evidences supported the existence of a major gene responsible for LDI activity [7,10]. Alignment of this region revealed 35 SNPs in the 150 accessions. Thirty-one SNPs (green-labeled in Figure 5) were significantly associated with LDI activity, explaining 13.4% of the phenotypic variation and –log_10_(p) value of each SNP reaching a highly significant level (Figure 5).

**Figure 6:**
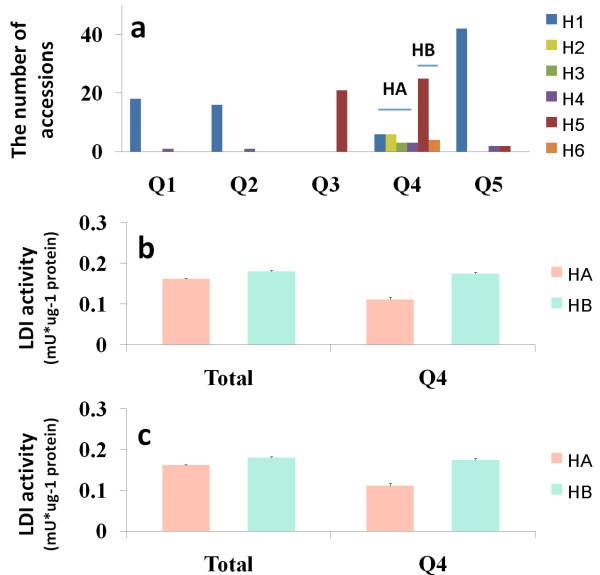
**The distribution and LDI activity of different haplotypes. (a)** The distribution of haplotypes among different subpopulations. **(b)** The LDI activity of HA and HB in total population and Q4 in 2011. **(c)** The LDI activity of HA and HB in total population and Q4 in 2012 HA includes H1, H2, H3 and H4; HB encompasses H5 and H6. Each bar is the mean of LDI activities of accessions belonging to the corresponding haplotypes. Error bars are SE values.

According to polymorphism of 35 SNPs, the 150 barley accessions can be divided into 6 haplotypes: H1-H6. H1-H4 showed high similarity of *HvLDI* sequence, while H5 and H6 had the similar sequences (Figure 5). Because the 31 significant SNPs in *HvLDI* were complete disequilibrium (r^2^ = 1) with each other, they could be regarded as one variant (Additional file 5: Figure S2). Hence, all accessions may be classified into two haplotypes, i.e. HA and HB. Accordingly, the distribution of haplotypes within different subpopulations was investigated. Q1, Q2 and Q5 were mainly constructed by haplotype 1, and Q3 by haplotype 5, whereas Q4 subpopulation showed specificity to other subpopulations for its balanced composition of HA and HB (HA: HB = 18:29). Interestingly, HA showed significantly lower LDI activity than HB in Q4 subpopulation (Figure 6b,c),while there was no difference between them in other subpopulations.

## Discussion

In this study, we investigated the genetic variation of LDI activity and performed GWAS analysis of *HvLDI*, in order to identify the loci controlling LDI activity in Tibetan wild barley. A wide variation in LDI activity was found in the 162 Tibetan wild barley accessions, and some of which displayed a minus value. It should be noted that some dextrins released by the de-branching enzymes during germination could also be detected by the Limit-Dextrizyme method, and these dextrins may account for the overestimation of LD activity [11,32,33]. Thus, the detected LD activities in extraction solutions of some accessions were higher than those of added LD, resulting in minus values of LDI activity, which was calculated as the reduced LD activity. However, the minus values of LDI in this experiment only occurred in very few accessions, and were very small (close to zero). Therefore the accessions with minus values of LDI are quite low in LDI activity. Actually, the impact of these dextrins produced during short-time extraction on the final LDI values could be negligible, as reported by [32].

Two DArT markers, i.e. bpb-8347 and bpb-0068 were found to be associated with LDI activities in Tibetan wild barley. Bpb-8347, a marker with highest GWA peak, is located near the locus of *HvLDI* gene obtained from IPK database, where the whole barley genome sequences lay [34]. This result confirms that *HvLDI* is a major gene controlling LDI activity in barley grain. Bpb-0068 might be a potential locus contributing to LDI activity, and it has not been reported up to date. Hence, it may be assumed that some minor effect genes affecting LDI activity may exist in Tibetan wild barley, and need to be confirmed by QTL analysis in segregating population in future work.

A total of 31 SNPs of *HvLDI* were significantly associated with LDI activity. It is also found that 11 of these SNPs are significantly associated with the malt extract in 56 cultivated genotypes in a previous study [19], in which association analysis between *HvLDI* gene and LDI activity was not conducted. These results indicate that the LDI activity has a close relationship with malt extract, because the two traits are affected by the same 11 SNPs. Indeed, it was previously reported that the *HvLDI* antisense lines showed unpredicted pleiotropic effects on numerous enzyme activities, including α- and β-amylases, starch synthases, as well as starch granule types, all of which affect malt extract [35]. In addition, the LD activity-associated SNP in the previous study [19] was not detected in the Tibetan wild population, suggesting that LD activity in malt may be independent on polymorphism of *HvLDI* gene in Tibetan population.

Interestingly, HA and HB showed a significant difference of the LDI activity only in Q4 subpopulation (Figure 6b,c). Q4 contains all kinds of haplotypes, while every other subpopulation mainly consists of one haplotype (Figure 6a). In other word, Q4 has a wider genetic diversity than every other subpopulation. It seems that these associated SNPs are false positive due to highly structured LD (Additional file 5: Figure S2) in this chromosomal region, and similar phenomenon were also observed in association analysis of frost tolerance [36,37]. It may be hypothesized that the variation of LDI activity in Q4 may be caused by other variations, which share linkage disequilibrium with the detected SNPs, in or around this region [34,38]. These variations should only belong to the accessions of HA in Q4 subpopulation, and may exist in the upstream or promoter region of *HvLDI*, or the regulated factors, which co-segregated with the detected SNPs. However, the assumption needs to be confirmed in the further work.

Four SNPs (13, 24, 72, and 352) within a coding region generate the alteration of the amino acids sequence. But these two kinds of haplotypes showed no significant difference in LDI activity among all accessions. It can be concluded that the alteration of LDI activity may be not due to the change of amino acid composition.

## Conclusions

This study confirmed the locus of LDI gene and detected a DArT marker, bpb-0068, associated with LDI activity. The results provide useful information for identifying minor effect genes affecting LDI activity, and also prove that Tibetan wild barley is an elite germplasm, which may act as an abundant gene pool for barley breeding. Furthermore, the detected SNPs in this study should be helpful for better understanding the genetic control of LDI activity in barley grain.

## Competing interests

The authors declare that they have no competing interests.

## Authors’ contributions

YH, GZ and CL designed the experiment, YH and SC determined the LDI activity, GWAS analysis and drafted the manuscript. LY and YH participated in the sequence alignment. DW and FD performed the data analysis. All authors read and approved the final manuscript.

## Supplementary Material

Additional file 1: Table S1 Minor allele frequency (MAF) and polymorphic information content (PIC) of 835 DArT markers used in this study. Click here for file

Additional file 2: Figure S1 Phylogenetic tree (neighbor-joining) of 162 barley accessions based on 835 DArT markers. Click here for file

Additional file 3: Table S2 The value of population structure of 162 accessions. Each accession belongs to the population with the highest value calculated by STRUCTURE software. Click here for file

Additional file 4: Table S3 GWAS results of bpb-8347 and bpb-0068 under Q, K, Q + K methods. Click here for file

Additional file 5: Figure S2 Decay of linkage disequilibrium (LD) of *HvLDI* gene in Tibetan wild barley. Each point in the LD matrix represents a comparison between a pair of polymorphic sites. Different colors represent different levels of LD. Click here for file
