# Supplementary figures and images for: Genetic architecture of limit dextrinase inhibitor (LDI) activity in Tibetan wild barley
Source: BMC Plant Biol. 2014 May 1;14:117. doi: 10.1186/1471-2229-14-117 (PMC4041910; doi:10.1186/1471-2229-14-117)

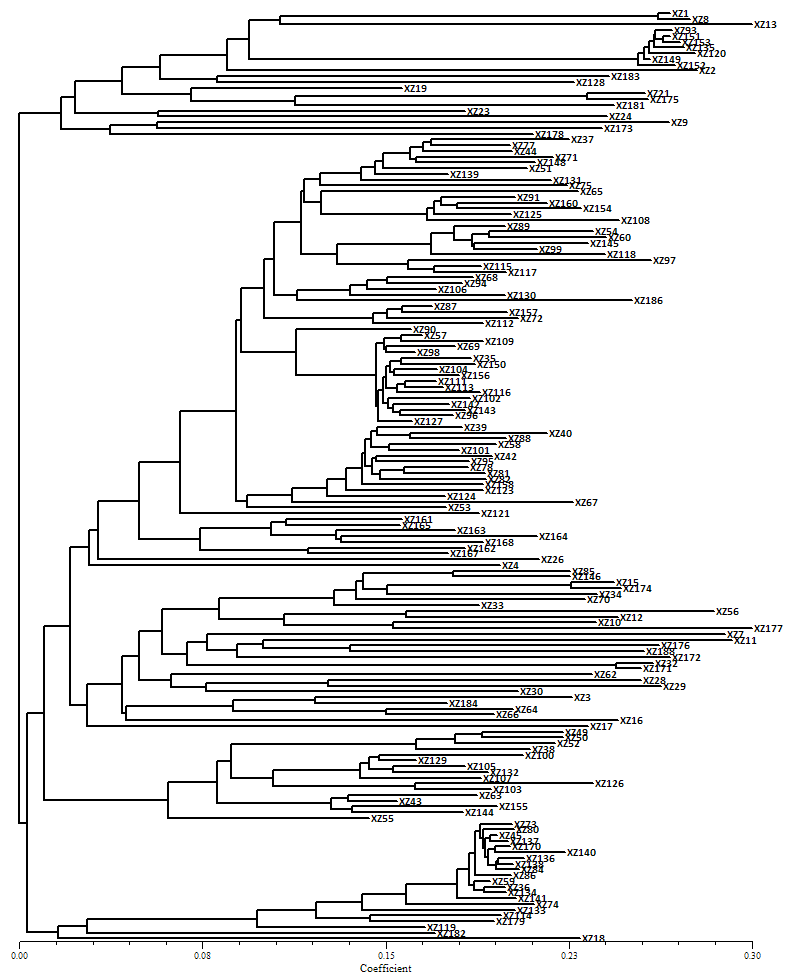

Supplement: Additional file 2: Figure S1 — Phylogenetic tree (neighbor-joining) of 162 barley accessions based on 835 DArT markers. [file 1471-2229-14-117-S2.tiff]

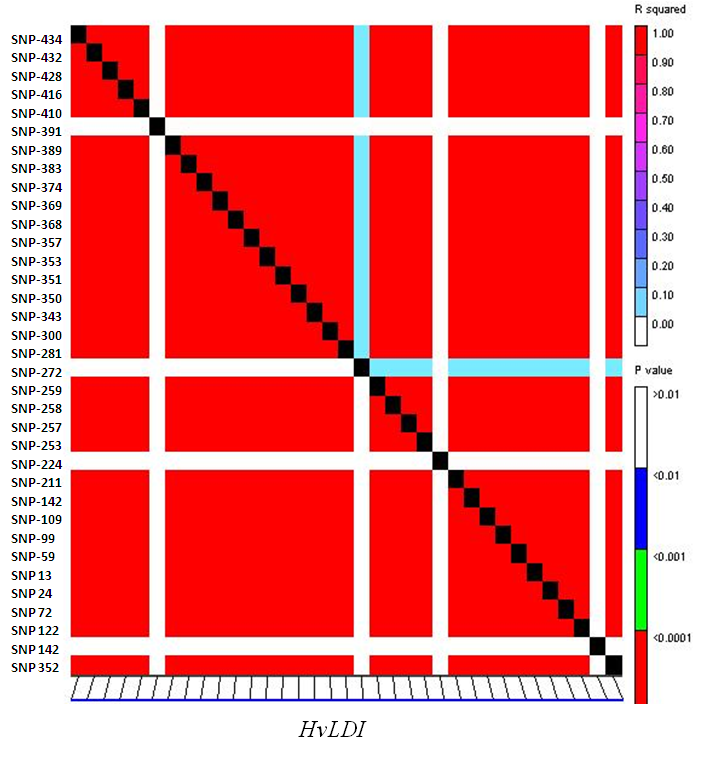

Supplement: Additional file 5: Figure S2 — Decay of linkage disequilibrium (LD) of HvLDI gene in Tibetan wild barley. Each point in the LD matrix represents a comparison between a pair of polymorphic sites. Different colors represent different levels of LD. [file 1471-2229-14-117-S5.tiff]
